# The effect of betel habits on blood glucose levels in the Karo ethnic community, Karo district

**DOI:** 10.12688/f1000research.75763.1

**Published:** 2022-01-26

**Authors:** Yunita Sari Pane, Yetty Machrina, Nurfida Khairina Arrasyid, Mutiara Indah Sari

**Affiliations:** 1 Department of Pharmacology and Therapeutics, Faculty of Medicine, Universitas Sumatera Utara, Medan, North Sumatra, 20155, Indonesia; 2 Department of Physiology, Faculty of Medicine, Universitas Sumatera Utara, Medan, North Sumatra, 20155, Indonesia; 3 Department of Parasitology, Faculty of Medicine, Universitas Sumatera Utara, Medan, North Sumatra, 20155, Indonesia; 4 Department of Biochemistry, Faculty of Medicine, Universitas Sumatera Utara, Medan, North Sumatra, 20155, Indonesia

**Keywords:** betel, gambir, T2DM, blood glucose levels, Batak-Karo ethnics

## Abstract

**Background:** Betel is a hereditary tradition from the ancestors of the Batak-Karo tribe, Indonesia. Karo people believe that betel is their unifier. The betel process begins with concocting a mixture of ingredients such as betel leaf, lime, gambier, areca nut, and with/without tobacco addition, then chewed slowly. Our previous study showed that gambier extracts ( *Uncaria gambier Roxb*), can reduce blood glucose levels (BGL) in type 2 diabetes mellitus (T2DM) patients. This study aimed to analyze whether the habit of chewing betel can affect BGL in the Karo ethnic community in the Karo district.

**Methods:** In total, 48 participants from the Karo community were divided into 4 groups (n=12 per group), namely: I. non-T2DM participants without betel habits; II. non-T2DM participants with betel habit; III. T2DM participants without betel habit and IV. T2DM participants with betel habit. The sampling technique was consecutive sampling. Data were collected by interviews and blood sampling (fasted and 2 hours postprandial (2hPP)). The collected data were analyzed by ANOVA then *post hoc* Bonferroni with a significance level of p-value <0.05.

**Results:** This study showed that fasting BGL had no difference in non-T2DM participants without betel habit (group-I) compared to non-T2DM participants with betel habit (group-II) (84.33±12.32 vs 81.00± 4,84), and T2DM participants without betel habit (group-III) compared to T2DM participants with betel habit (group-IV) (196.25± 104.81 vs 150.00 ± 42.45), p>0.05. On the other hand, there was a significant difference in BGL 2hPP in group T2DM participants with betel habit compared to all groups (p<0.05). T2DM participants without betel habit group had the highest BGL levels compared to other groups.

**Conclusions:** This study concluded that the habit of chewing betel containing gambier is effective in restraining the rate of increase in blood glucose levels. Further research is needed to see the mechanism.

## Introduction

Betel is a cultural tradition of Indonesian society with one of its compositions being gambier. Gambier ( *Uncaria gambier Roxb*) is mixed with several other ingredients then wrapped in betel leaf which is then chewed slowly. People who chew betel regularly have their own reasons why they have the habit, other than taste. Chewing is a hereditary tradition from ancestors in the Karo tribe. The Karo people believe that betel is a unifying activity for them ( Perangin-angin, 2017).

The habit of betel is usually done 3 times a day, namely in the morning, after lunch and at night ( Kanapathy, 2014). The habit of betel is mostly seen in women of the Karo tribe, but there are also men who do it, because chewing betel is always done when meeting with relatives, colleagues or in other social settings ( Sinuhaji, 2010). According to data derived from interviews, chewing betel provides benefits, namely to enjoy pleasures such as smoking, for leisure, and to eliminate bad breath. Chewing betel has been done for generations and because of the belief that this activity can strengthen teeth ( Flora *et al.*, 2012) as well as maintain health.

Chewing betel has been a habit in society for a long time, but nowadays we rarely encounter it, because there has been a shift in values, even though in rural areas there are still many habits that must be participated in because they uphold the traditions of generations ( Perangin-angin, 2017). Chewing betel is done in different ways from one country to another and from one region to another in the same country ( Gupta & Ray, 2004). However, the composition of betel is relatively consistent, consisting of betel leaf, betel nut (Areca Catechu), lime (calcium hydroxide) and gambier ( *Uncaria gambier Roxb*) ( Lombu, 2014).

The content of catechin polyphenols in gambier is efficacious as an anti-oxidant that may prevent various diseases, such as diabetes mellitus ( Umeno *et al.*, 2016). This is evidenced in the research of Pane *et al*. (2018) which states that gambier extract is efficacious in the treatment of diabetes by increasing levels of superoxide dismutase and lowering blood glucose levels (BGL).

From the description above, the researchers wanted to assess whether the habit of chewing betel can affect BGL in subjects with T2DM compared to participants without T2DM, studied in the Batak-Karo tribe in Karo District.

## Methods

### Ethical considerations

This research obtained ethical approval from the Health Research Ethics Committee of the University of North Sumatra (No. 468/KEP/USU/2021). Participants gave written informed consent after receiving an explanation from the researcher regarding the research procedure they would undergo ( Pane *et al.*, 2022).

### Participants

The sample size was estimated following data from Kawamori *et al.* (2014) for BGL fasting and BGL 2hPP, using the following formula:

* Calculation for sample size of BGL fasting

$$\begin{array}{c} \sigma^{2}=\frac{(\text{n}1-1)S1^{2}+(\text{n}2-1)S2^{2}}{\left(n1+n2-2\right)}\\ \sigma^{2}=\frac{(88-1)107^{2}+(33-1)77^{2}}{\left(88+33-2\right)}\\ \sigma^{2}=\frac{996,063+189,728}{119}\\ \sigma =\sqrt{9,964.630}\\ \sigma =99.82 \end{array}$$

$$\text{n}=\frac{2\sigma^{2}{\left(Z_{\left(1-\frac{\alpha }{2}\right)}+Z_{\left(1-\beta \right)}\right)}^{2}}{{\left(\text{μ}1-\text{μ}2\right)}^{2}}$$

$$\text{n}=2{\left(99.82\right)}^{2}\;\frac{{\left(1.64+0.842\right)}^{2}}{(\text{−}134-{\left(-14\right)}^{2}}$$

$$\text{n}=2(9,964.03)\;\frac{{\left(2,482\right)}^{2}}{{\left(\text{−}120\right)}^{2}}$$

$$\text{n}=2(9,964.03)\;\frac{\left(6.160\right)}{\left(14,400\right)}$$

n=2(9,964.03)(0.000428)

n=8.53

n≈9participants.

* Calculation for sample size of BGL 2hPP

$$\begin{array}{c} \sigma^{2}=\frac{(\text{n}1-1)S1^{2}+(\text{n}2-1)S2^{2}}{\left(n1+n2-2\right)}\\ \sigma^{2}=\frac{(88-1)43.6^{2}+(33-1)39.8^{2}}{\left(88+33-2\right)}\\ \sigma^{2}=\frac{(87)\;1,900.96+(32)\;1,584.04}{\left(119\right)}\\ \sigma^{2}=\frac{165,383.52+50,689.28}{119}\\ \sigma =\sqrt{1,815.74}\\ \sigma =42.61 \end{array}$$

$$\text{n}=2\sigma^{2}\;\frac{{\left(z\alpha +z\beta \right)}^{2}}{{\left(\text{μ}1-\text{μ}2\right)}^{2}}$$

$$\text{n}=2{\left(42.61\right)}^{2}\;\frac{{\left(1.64+0.842\right)}^{2}}{(\text{−}50.2-{\left(\text{−}4.2\right)}^{2}}$$

$$\text{n}=2\left(1,815.61\right)\;\frac{{\left(1.64+0.842\right)}^{2}}{(\text{−}50.2-{\left(\text{−}4.2\right)}^{2}}$$

$$\text{n}=2(1,815.61)\;\frac{6.160}{2,116}$$

n=2(1,815.61)(0.0029)

n=10.53participants

n≈11participants.

The calculation of the number of samples in the fasting BGL group is minimal 9 participants, while in the 2hPP BGL group minimal 11 participants.

The number of samples used in this study was taken from the largest number which calculated based on sample size formula from the two groups (BGL fasting or BGL 2hPP). The largest number of samples was taken from the BGL 2hPP group i.e: 11 participants. However, to anticipate the possibility of dropped out, the number of participants is added by 10% from a total of 11 participants = 12 people.

So from the 4 groups studied each consisted of 12 participants.

σ ^2^=population variance; σ=population standard deviation (SD); n=total number of samples each group (n _1_=88; n _2_=33); $Z_{\left(1-\frac{\alpha }{2}\right)}$ = value in the standard normal distribution equal to the level of significance α = 1.64; Z _(1-β)_= value in the standard normal distribution equal to the desired, β= 0.842; µ _1_=mean outcome exposed/treatment group (-134 mg/dl) for BGL fasting and (-50.2 mg/dl) for BGL 2 hPP); µ _2_=mean outcome unexposed/placebo group (-14 mg/dl) for fasting BGL and (-4.2 mg/dl) for 2hPP BGL; S _1_=SD changes from baseline treatment group (107 mg/dl) for fasting BGL and (43.6 mg/dl) for 2hPP BGL; S _2_=SD changes from baseline placebo group (77 mg/dl) for fasting BGL and (39.8 mg/dl) for 2hPP BGL. (see data from Table 2 in Kawamori *et al.*, 2014).

Participants were divided into four groups based on feedback from the questionnaire regarding their betel habits: Group-I. Non T2DM participants without betel habit; II. Non T2DMparticipants with betel habit; III. T2DMparticipants without betel habit and IV. T2DMparticipants with betel habit. Inclusion criteria were participants with 2 generations of the native Karo Batak tribe, aged between 20–70 years. Exclusion criteria were subjects who have a chronic disease (complication), such as liver disease, kidney disease, cardiovascular disease, lung disease, etc, and T2DM participants on insulin therapy.

The sampling technique used was consecutive sampling. The prospective participants were approached to join the study by conducting a survey previously to the research location to see the betel habits of the Karo people. After that, the research team visited the local health service center (Puskesmas Dolat Rayat) to find out about health data and the betel habits of the local community. Then assisted by Puskesmas staff in collecting participants. Then a time was determined at which the research team met directly and gave an explanation of the aims and objectives of the researcher to research the local community. The research team explained the benefits of betel habits for health, especially to reduce blood glucose levels in T2DM patients because of the presence of gambier ( *Uncaria gambier Roxb*) as one of the components in betel which has an antioxidant effect that can reduce BGL.

### Data collection

This research was conducted from July 2021 to October 2021 at the Puskesmas Dolat Rayat Karo District. Data collection of the betel habits of the subjects in this study was done via questionnaire and blood sampling to measure data BGL. These were done on the same day. The questionnaire asked for the following information: name, age, education, occupation, history of illness, history of medicine, family history of illness, betel habit.

Before taking blood, subjects were asked to fast (at home) from 10. ^00 ^pm to 08. ^00^ am (around 10 hours) the next day. Blood samples were taken at 08. ^00^ am straight after fasting (fasting BGL). Following this, the subjects consumed 100 grams of white bread, and 2 hours later the blood was taken again (BGL 2h postprandial [PP]).

Blood was drawn from the participant’s fingertip and BGL level checked using a glucometer (Family Dr® Blood Glucose Monitoring System, AGM-513S, All Medicus Co., Ltd.). The following steps were taken:

1. Wash the participant’s hands, use an alcohol swab. Make sure the finger are dry before testing.
2. Insert a test strip into the glucometer.
3. Set up the lancing device, unscrew the cap on the lancing device. Insert the lancet into the lancing device, then twist off the cap on the lancet, recap the lancing device by screwing the top back on next set the depth of the lancing device
4. Slide the cocking handle back
5. Hold the finger and place the lancing device firmly on the side of the finger and push the button
6. Squeeze from the palm of the hand down to the tip of the finger to obtain a drop of blood
7. Place the blood sample on the test strip
8. The glucometer does the assessment and gives the result

### Statistical analysis

The data collected were analyzed using SPSS version 20 by ANOVA and *post hoc* Bonferroni test with a significance value of p <0.05.

## Results

The participants in this study were the Karo tribe from 2 generations of pure Karo natives. Of 49 potential participants, 48 were eligible to take part ( Pane *et al.*, 2022). The characteristics of the distribution of participants based on betel habits, as follows:

Table 1 shows there were 12 participants in each of the 4 groups studied. It was found that 14 participants (29,17%) in the betel habit groups had a frequency of betel >10 times a day, and the duration of betel habit as more than 10 years compared to less than 10 years was equal (12 participants, 25% in each group). Most participants (16 people; 33,33 %) reported benefits of betel to maintain health, especially mind relaxation (mood) and extra benefit for healthy and strong teeth, while others had no special reason for using betel, only to follow their customs (8 people; 16,67%). In the T2DM group, there were 21 participants (43,75%) who had chewed betel suffering from diabetes for <10 years. The participants included 11 men (22, 92 %) and 37 women (77,08 %). The most populous age group was 51–60 years (19 participants; 33,3 %) and least was 20–30 years (5 participants; 10,42 %). The most populous BMI category was the normal-weight group (19 participants; 39,58%). The BMI group below normal weight included 1 person (2,08%). The other participants were above normal weight. BMI classification was based on WHO (2021). Most participants had at least Senior High School level education (26 participant; 54,17%). Some worked as farmers (14 participants; 29,17%) but the largest number of participants were housewives (15 participants; 31,25%).

**Table 1.** Distribution of respondents based on the betel chewing habit of the Batak-Karo tribe in Karo District. T2DM=type 2 diabetes mellitus.

	n	Percentage (%)
**Study group** - Non T2DM without betel habit - Non T2DM with betel habit - T2DM without betel habit - T2DM with betel habit	12 12 12 12	25 25 25 25
**Gender** - Men - Women	11 37	22.92 77.08
**Age** - 20–30 - 31–40 - 41–50 - 51–60 - 61–70	5 9 9 16 9	10.42 18.75 18.75 33.33 18.75
**Body mass index** - Under weight - Normal weight - Overweight - Obese Class-I - Obese Class-II - Obese Class-III	1 19 17 6 4 1	2.08 39.58 35.42 12.5 8.33 2.08
**Duration of T2DM** - >10 years - <10 years - Never	3 21 24	6.25 43.75 50
**Betel Frequency** - Often >10 x in daily - Sometimes/rarely < 10 x in daily - Never	14 10 24	29.17 20.83 50
**Duration of Betel habit** - >10 years - <10 years - Never	12 12 24	25 25 50
**Aims of betel** - Calms the mind (mood), make the teeth be healthy and strong - Only as habit/tradition - Never	16 8 24	33.33 16.67 50
**Effects of betel** (felt comfort, change coloring teeth, fresh condition feeling, irritation in mouth, red lips, etc) - Yes - No	24 24	50 50
**Education** - No School - Elementary school - Junior high school - Senior high school - Academic - Undergraduate - Magister	2 5 5 26 4 5 1	4.17 10.42 10.42 54.17 8.33 10.42 2.08
**Occupation** - Retired/pension - Housewife/no work - Entrepreneur/sales - Civil employee - Private employee - Farmer	1 15 9 5 4 14	2.08 31.25 18.75 10.42 8.33 29.17

Table 2 shows the BGL of participants based on with/without betel habits in the non- T2DM and T2DM groups.

**Table 2.** Blood glucose levels (BGL) after fasting and 2 hours postprandial (2hPP). T2DM=type 2 diabetes mellitus.

Groups	BGLfasting (mg/dl)	BGL 2hPP (mg/dl)	Gap of BGL (mg/dl)
I. Non-T2DM participants without betel habit	84.33 ± 12.32	111.25 ± 22.62	26.92
II. Non-T2DM participants with betel habit	81.00 ± 4.84	108.33 ± 18.99	27.33
III.T2DM participants without betel habit	196.25 ± 104.81	314.92 ± 128.97	118.67
IV.T2DM participants with betel habit	150.00 ± 42.45	229.25 ± 58.26	79.25

This study showed that for fasting BGL there was no significant difference between group I vs -II (84.33 ± 12.32 vs 81.00 ± 4.84; p = 1,000), and group-III compared to group-IV (196.25 ± 104.81 vs 150.00 ± 42.45; p=0.317). There was no significant difference in BGL between the comparison of the betel and the no-betel groups. However, there was a difference between group -I compared to group III (84.33 ± 12.32 vs 196.25 ± 104.81; p=0.000) and groups I-IV (84.33 ± 12.32 vs 150.00 ± 42.45; p=0.042) which shows the significant difference of BGL. Similarly, group-II was compared with group-III (81.00 ± 4.84 vs 196.25 ± 104.81; p=0.000) and group-II was compared to group-IV (81.00 ± 4.84 vs 150.00 ± 42.45; p=0.029) showing statistically significant differences in fasting BGL (see Table 2).

In BGL 2hPP, there was no significantly difference between group-I vs -II (112.25 ± 22.62 vs 108.33 ± 18.99; p = 1,000). On the other hand, comparisons of all other groups showed significant differences in BGL, such as: group-I compared group-III (112.25 ± 22.62 vs 314.92 ± 128.97 mg/dl) (p=0.000); group-I compared group-IV (112.25 ± 22.62 vs 229.25 ± 58.26 mg/dl) (p=0.001); group-II compared group-III (108.33 ± 18.99 vs 314.92 ± 128.97 mg/dl) (p=0.000), group-II compared group-IV (108.33 ± 18.99 vs 229.25 ± 58.26 mg/dl) (p=0.001) and group-III compared group-IV (314.92 ± 128.97 vs 229.25 ± 58.26 mg/dl) (p=0.035) ( Table 2).

Table 2 shows the differences in a gap of BGL in each group and the magnitude of the increase in BGL fasting compared to BGL 2 hours postprandial. The difference in the increase in BGL groups I and II was almost the same, namely 26.92 and 27.33 mg/dl, not exceeding normal glucose levels in both fasting and 2 hours postprandial conditions with/without habit betel. But the highest BGL difference was found in group III-T2DM without betel habits, which was 118.67 mg/dl. Meanwhile, in the T2DM with betel habits (group-IV), the gap between BGL fasting and BGL2hPP was only 79.25 mg/dl. This indicates that the betel habit can restrain the increase in BGL as seen in groups IV compared to group III.

## Discussion

The frequency of betel habit among participants in this study varied. In two groups (-II and -IV), 14 participants from a total of 24 participants in those groups had betel habits > 10 times a day (29.17%). The results of this study are the same as Kanapathy's study (2014), in which 14 samples of a total of 25 had betel habit frequency > 10 times a day.

In the present study, the highest percentage of participants suffering from Diabetes Mellitus (DM) for less than 10 years was 21 participants (43.75%), in contrast to the findings of Budiharto (2018), who found that 15 out of 25 people (60%) who had suffered from DM long-term and had a l betel habit for more than 10 years. The main purpose of betel habits found in this study (16 participants, 33.33%) was to get a sense of comfort and dental health. This is supported by Budiharto's research (2018) which reported that out of a total of 25 participants (13 participants, 52%) the habit of chewing betel had the same effect. In contrast, the results of other researchers showed that as many as 68% of participants experienced porous teeth and poor oral hygiene due to betel. This could be because the subjects studied did not maintain oral hygiene, or lacked the knowledge about how to maintain oral health by chewing betel ( Andriyani, 2005).

The habit of chewing betel is often found in rural areas in Karo Regency. Chewing betel is a hereditary culture that has become a tradition of the Karo tribe to this day. This is supported by research conducted by Perangin-Angin (2017) which states that the Karo people have a tradition that involves betel activities in a series of Karo customs. However, unlike the Karo people in the countryside, the Karo people in urban areas are rarely found to have the habit of chewing betel. This may be due to hygiene factors (when they chew betel, their teeth change to turn blackish red, and also differences in busy urban lifestyles whereas Karo people who live in urban areas do not have much time to gather while chewing betel together).

Betel habit generally uses a mixture of betel leaf, lime (calcium hydroxide), gambier, areca nut, sometimes with or without the addition of tobacco. Gambier is known to prevent various diseases because besides being efficacious as an anti-inflammatory, it is also a strong anti-oxidant. Pane *et al*. (2018) reported that gambier can reduce blood glucose levels in T2DM patients by increasing levels of superoxide dismutase resulting in a decrease in malondialdehyde formation and an increase in pancreatic function in producing insulin. In the present study, we suggested that the habit of chewing betel can control BGL because of the efficacy of gambier which is one of the components in betel. It was proved in the results that there were differences in BGL in each group with an increase in the rate of fasting BGL and 2 hours postprandial BGL which can be compared as follows: The T2DM group with betel habits (-IV) had a lower difference in the increase in BGL (79.25) mg/dl compared to the T2DM group without betel (-III; 118.67) mg/dl. In the group that has the habit of consuming betel, glucose levels are lower than the group that does not have the habit of consuming betel. We assumed that gambier consumed as part of their betel habit has the ability to bind oxidants produced by metabolism when blood glucose levels are high, thus affecting the function of the pancreas to produce insulin. This therefore causes a suppressed rate of increase in BGL in participants who are suffering from T2DM with betel habit. However, in the non-T2DM group (-I and -II), both groups with or without betel habits had BGL within normal limits. Gambier ( *Uncaria gambier Roxb*) which is rich in catechins plays a role in the normalization of BGL ( Sugiyama, 2005). Most importantly, the anti-oxidant catechin molecules in gambier are safe, which were identified as the main bioactive compounds in gambier ( Anggraini *et al.*, 2011). Catechins can improve diabetes and its complications by modifying oxidative stress (p<0.05) ( Pane *et al.*, 2018; Samarghandian *et al.*, 2017).

Comparing the fasting BGL between groups, there was no statistically significant difference between group non T2DM participants without betel habit (-I) and non T2DM participants with betel habit (-II); and also group T2DM participants without betel habit(-III) compared T2DM participants with betel habit (-IV), p>0.05. However, there were statistically significant differences between BGL in group-III and group-I and group-III and group-II; group-IV and group-I; group-IV and group-III, p<0.05.

While the comparison of BGL 2 hours postprandial showed a significant difference between each group (p<0.05), except for the comparison of BGL group I and group II there was no significant difference (p=1,000).

The difference between fasting BGL and 2hPP was found in the non-betel (-III) T2DM group which had the highest BGL compared to the other groups. Similarly, the comparison of the largest differences in the increase in BGL rates was shown in group III. We assume that in group III there is no gambier to suppress BGL levels, in contrast to the group that has the habit of chewing betel, it seems that BGL levels are restrained. The comparison of fasting BGL and 2hPP in group with betel habit (-II and -IV) was lower than the group without betel habit (-I and -III). Another interesting thing in this study showed that there was no suppression of BLG below the normal threshold in BGL fasting and BGL 2hPP condition in the group of non T2DMparticipants with betel habit (group-II). The limit of normal BGL = 72 -108 mh/dL ( Mathew & Tadi, 2021).

The limitations of this study were that it is not easy to collect samples that have betel habits such as groups-II and -IV, due to the small number of samples in the population. In addition, in a pandemic situation, people do not want to be at in the Public Health Center for long, especially for T2DM with/without betel habit groups. However, every step in this study was carried out under strict health protocol procedures.

## Conclusion

This study concluded that betel habits can restrain the increase in BGL as seen in a comparison of T2DM participants with betel habit (group-IV) compared to T2DM participants without betel habit (group-III). But this wasn’t the case when comparing participants without T2DM.

## Data availability

### Underlying data

Figshare: The Effect of Betel Habits on Blood Glucose Levels in Karo ethnic community in Karo District. https://doi.org/10.6084/m9.figshare.17871911 ( Pane *et al.*, 2022).

This project contains the following underlying data:

- 2021-data.xlsx (raw data in Indonesian)
- Output.xlsx (statistical analysis)

### Extended data

Figshare: The Effect of Betel Habits on Blood Glucose Levels in Karo ethnic community in Karo District. https://doi.org/10.6084/m9.figshare.17871911 ( Pane *et al.*, 2022).

This project contains the following extended data:

- Informed Consent.pdf
- Certificate Clinical Trial Yunita Sari Pane.pdf
- ethical clearance.pdf
- QUESIONER PENELITIAN-20122021.docx (questionnaire in Indonesian)
- Lampiran-sign.docx (information sheet in Indonesian)

Data are available under the terms of the Creative Commons Zero "No rights reserved" data waiver (CC0 1.0 Public domain dedication).
