# The effect of betel habits on blood glucose levels in the Karo ethnic community, Karo district

**DOI:** 10.12688/f1000research.75763.2

**Published:** 2022-08-18

**Authors:** Yunita Sari Pane, Yetty Machrina, Nurfida Khairina Arrasyid, Mutiara Indah Sari

**Affiliations:** 1 Department of Pharmacology and Therapeutics, Faculty of Medicine, Universitas Sumatera Utara, Medan, North Sumatra, 20155, Indonesia; 2 Department of Physiology, Faculty of Medicine, Universitas Sumatera Utara, Medan, North Sumatra, 20155, Indonesia; 3 Department of Parasitology, Faculty of Medicine, Universitas Sumatera Utara, Medan, North Sumatra, 20155, Indonesia; 4 Department of Biochemistry, Faculty of Medicine, Universitas Sumatera Utara, Medan, North Sumatra, 20155, Indonesia

**Keywords:** betel, gambir, T2DM, blood glucose levels, Batak-Karo ethnics

## Abstract

**Background:** Betel habit is a hereditary tradition from the ancestors of the Batak-Karo tribe, Indonesia. Karo people believe that betel is their unifier. The betel process begins with concocting a mixture of ingredients such as betel leaf, lime, gambier, areca nut, and with/without tobacco addition, then chewed slowly. Our previous study showed that gambier extracts ( *Uncaria gambier Roxb*), can reduce blood glucose levels (BGL) in type 2 diabetes mellitus (T2DM) patients. This study aimed to analyze whether the habit of chewing betel can affect BGL in the Karo ethnic community in the Karo district.

**Methods:** In total, 48 participants from the Karo community were divided into 4 groups (n=12 per group), namely: I. non-T2DM participants without betel habits; II. non-T2DM participants with betel habit; III. T2DM participants without betel habit and IV. T2DM participants with betel habit. The sampling technique was consecutive sampling. Data were collected by interviews and blood sampling (fasted and 2 hours postprandial (2hPP)). The collected data analyzed by paired *t-test*, Independent sample *t-test*, Wilcoxon, and Mann-Whitney with a significance level of p-value <0.05.

**Results:** This study showed that fasting BGL condition in group-I compared to group-II (84.33±12.32 vs 81.00±4.84)mg/dl; and group-III compared to group-IV (196.25±104.81 vs 150.00±42.45)mg/dl had no significant difference. Also, the BGL of 2hPP condition in group-I compared to group-II (111.25 ±22.62 vs 108.33±18.99) mg/dl, and group-III compared to group-IV (314.92±128.97 vs 229.25±58.26) mg/dl, in statistically there was no difference (p>0.05). Although the data showed that group-III was higher than group-IV.

**Conclusions:** This study concludes that with/without betel habits affect blood sugar levels in type 2 diabetes subjects (Groups III and IV).

## Introduction

Betel habit is a cultural tradition of Indonesian society with one of its compositions being gambier. Gambier ( *Uncaria gambier Roxb*) is mixed with several other ingredients then wrapped in betel leaf which is then chewed slowly. People who chew betel regularly have their own reasons why they have the habit, other than taste. Chewing betel is a hereditary tradition from ancestors in the Karo tribe. The Karo people believe that betel is a unifying activity for them ( Perangin-angin, 2017).

The habit of betel is usually done 3 times a day, namely in the morning, after lunch and at night ( Kanapathy, 2014). The habit of betel is mostly seen in women of the Karo tribe, but there are also men who do it, because chewing betel is always done when meeting with relatives, colleagues or in other social settings ( Sinuhaji, 2010). According to data derived from interviews, chewing betel provides benefits, namely to get pleasures such as smoking, for leisure, and to eliminate bad breath. Chewing betel has been done for generations and because of the belief that this activity can strengthen teeth ( Flora *et al.*, 2012) as well as maintain health.

Chewing betel has been a habit in society for a long time, but nowadays we rarely encounter it, because there has been a shift in values, even though in rural areas there are still many habits that must be participated in because they uphold the traditions of generations ( Perangin-angin, 2017). Chewing betel is done in different ways from one country to another and from one region to another in the same country ( Gupta & Ray, 2004). However, the composition of betel is relatively consistent, consisting of betel leaf, betel nut (Areca Catechu), lime (calcium hydroxide) and gambier ( *Uncaria gambier Roxb*) ( Lombu, 2014).

The content of catechin polyphenols in gambier is efficacious as an anti-oxidant that may prevent various diseases, such as diabetes mellitus ( Umeno *et al.*, 2016). This is evidenced in the research of Pane *et al*. (2018) which states that gambier extract is efficacious in the treatment of diabetes by increasing levels of superoxide dismutase and lowering blood glucose levels (BGL).

From the description above, the researchers wanted to assess whether the habit of chewing betel can affect BGL in subjects with T2DM compared to participants without T2DM, studied in the Batak-Karo tribe in Karo District.

## Methods

### Ethical considerations

This research obtained ethical approval from the Health Research Ethics Committee of the Universitas Sumatera Utara (No. 468/KEP/USU/2021). Participants gave written informed consent after receiving an explanation from the researcher regarding the research procedure they would undergo ( Pane *et al.*, 2022).

### Participants

The sample size was estimated following data from Kawamori *et al.* (2014) for BGL fasting and BGL 2hPP, using the following formula:

* Calculation for sample size of BGL fasting

$$\begin{array}{c} \sigma^{2}=\frac{(\text{n}1-1)S1^{2}+(\text{n}2-1)S2^{2}}{\left(n1+n2-2\right)}\\ \sigma^{2}=\frac{(88-1)107^{2}+(33-1)77^{2}}{\left(88+33-2\right)}\\ \sigma^{2}=\frac{996,063+189,728}{119}\\ \sigma =\sqrt{9,964.630}\\ \sigma =99.82 \end{array}$$

$$\text{n}=\frac{2\sigma^{2}{\left(Z_{\left(1-\frac{\alpha }{2}\right)}+Z_{\left(1-\beta \right)}\right)}^{2}}{{\left(\text{μ}1-\text{μ}2\right)}^{2}}$$

$$\text{n}=2{\left(99.82\right)}^{2}\;\frac{{\left(1.64+0.842\right)}^{2}}{(\text{−}134-{\left(-14\right)}^{2}}$$

$$\text{n}=2(9,964.03)\;\frac{{\left(2,482\right)}^{2}}{{\left(\text{−}120\right)}^{2}}$$

$$\text{n}=2(9,964.03)\;\frac{\left(6.160\right)}{\left(14,400\right)}$$

n=2(9,964.03)(0.000428)

n=8.53

n≈9participants.

* Calculation for sample size of BGL 2hPP

$$\begin{array}{c} \sigma^{2}=\frac{(\text{n}1-1)S1^{2}+(\text{n}2-1)S2^{2}}{\left(n1+n2-2\right)}\\ \sigma^{2}=\frac{(88-1)43.6^{2}+(33-1)39.8^{2}}{\left(88+33-2\right)}\\ \sigma^{2}=\frac{(87)\;1,900.96+(32)\;1,584.04}{\left(119\right)}\\ \sigma^{2}=\frac{165,383.52+50,689.28}{119}\\ \sigma =\sqrt{1,815.74}\\ \sigma =42.61 \end{array}$$

$$\text{n}=2\sigma^{2}\;\frac{{\left(z\alpha +z\beta \right)}^{2}}{{\left(\text{μ}1-\text{μ}2\right)}^{2}}$$

$$\text{n}=2{\left(42.61\right)}^{2}\;\frac{{\left(1.64+0.842\right)}^{2}}{(\text{−}50.2-{\left(\text{−}4.2\right)}^{2}}$$

$$\text{n}=2\left(1,815.61\right)\;\frac{{\left(1.64+0.842\right)}^{2}}{(\text{−}50.2-{\left(\text{−}4.2\right)}^{2}}$$

$$\text{n}=2(1,815.61)\;\frac{6.160}{2,116}$$

n=2(1,815.61)(0.0029)

n=10.53participants

n≈11participants.

The calculation of the number of samples in the fasting BGL group is minimal 9 participants, while in the 2hPP BGL group minimal 11 participants.

The number of samples used in this study was taken from the largest number which calculated based on sample size formula from the two groups (BGL fasting or BGL 2hPP). The largest number of samples was taken from the BGL 2hPP group i.e: 11 participants. However, to anticipate the possibility of dropped out, the number of participants is added by 10% from a total of 11 participants ≈ 12 people.

So from the 4 groups studied each consisted of 12 participants.

σ ^2^=population variance; σ=population standard deviation (SD); n=total number of samples each group (n _1_=88; n _2_=33); $Z_{\left(1-\frac{\alpha }{2}\right)}$ = value in the standard normal distribution equal to the level of significance α = 1.64; Z _(1-β)_= value in the standard normal distribution equal to the desired, β= 0.842; µ _1_=mean outcome exposed/treatment group (-134 mg/dl) for BGL fasting and (-50.2 mg/dl) for BGL 2 hPP); µ _2_=mean outcome unexposed/placebo group (-14 mg/dl) for fasting BGL and (-4.2 mg/dl) for 2hPP BGL; S _1_=SD changes from baseline treatment group (107 mg/dl) for fasting BGL and (43.6 mg/dl) for 2hPP BGL; S _2_=SD changes from baseline placebo group (77 mg/dl) for fasting BGL and (39.8 mg/dl) for 2hPP BGL. (see data from Table 2 in Kawamori *et al.*, 2014).

Participants were divided into four groups based on feedback from the questionnaire regarding their betel habits: Group-I. Non T2DM participants without betel habit; II. Non T2DMparticipants with betel habit; III. T2DMparticipants without betel habit and IV. T2DM participants with betel habit.

**Inclusion criteria**

Participants were 2 generations of the native Karo Batak tribe, aged between 20–70 years.

**Exclusion criteria**

Participants were subjects who have a chronic disease (complication), such as liver disease, kidney disease, cardiovascular disease, lung disease, T2DM on insulin therapy,

The sampling technique used was consecutive sampling. The prospective participants were approached to join the study by conducting a survey previously to the research location to see the betel habits of the Karo people. After that, the research team visited the local health service center (Puskesmas Dolat Rayat) to find out about health data and the betel habits of the local community. Then assisted by Puskesmas staff in collecting participants. Then a time was determined at which the research team met directly and gave an explanation of the aims and objectives of the researcher to research the local community. The research team explained the benefits of betel habits for health, especially to reduce blood glucose levels in T2DM patients because of the presence of gambier ( *Uncaria gambier Roxb*) as one of the components in betel which has an antioxidant effect that can reduce BGL.

### Data collection

This research was conducted from July 2021 to October 2021 at the Puskesmas Dolat Rayat Karo District. Data collection of the betel habits of the subjects in this study was done via questionnaire and blood sampling to measure data BGL. These were done on the same day. The questionnaire asked for the following information: name, age, education, occupation, history of illness, history of medicine, family history of illness, betel habit.

Before taking blood, subjects were asked to fast (at home) from 10. ^00^ pm to 08. ^00^ am (around 10 hours) the next day. Blood samples were taken at 08. ^00^ am straight after fasting (fasting BGL). Following this, the subjects consumed 100 grams of white bread, and 2 hours later the blood was taken again (BGL 2h postprandial [PP]).

### Procedures took blood for measured Blood Glucose Levels

Blood was drawn from the participant’s fingertip and the BGL was checked using a glucometer (Family Dr® Blood Glucose Monitoring System, AGM-5135, All Medicus Co., Ltd.). The steps taken are starting with washing hands and using an alcohol swab. After the subject's finger was dry, the test strip was inserted into the glucometer. The lancing device is prepared and open the lid on the device. The lancet is inserted into the lancing device by unscrew the top back and adjusting the depth of the lancing device. The cocking handle is shifted to the rear. Then the finger being examined is placed firmly on the side of the other finger and the lancing device button is pressed. After that, the finger is squeezed from the palm to the fingertips to get a drop of blood. The prepared test strip is then attached to the blood sample and the glucometer will show the test value.

### Statistical analysis

The data collected were analyzed using SPSS version 20 by paired *t-test*, Independent sample *t-test*, Wilcoxon, and Mann-Whitney with a significance value of p <0.05.

## Results

The participants in this study were the Karo tribe from 2 generations of pure Karo natives. Of 49 potential participants, 48 were eligible to take part ( Pane *et al.*, 2022). The characteristics of the distribution of participants based on betel habits, as follows:

Table 1 shows there were 12 participants in each of the 4 groups studied. It was found that 14 participants (29.17%) in the betel habit groups had a frequency of betel >10 times a day, and the duration of betel habit as more than 10 years compared to less than 10 years was equal (12 participants, 25% in each group). Most participants (16 people; 33.33 %) reported benefits of betel to maintain health, especially mind relaxation (mood) and extra benefit for healthy and strong teeth, while others had no special reason for using betel, only to follow their customs (8 people; 16.67%). In the T2DM group, there were 21 participants (43.75%) who had chewed betel suffering from diabetes for <10 years. The participants included 11 men (22.92 %) and 37 women (77.08 %). The most populous age group was 51–60 years (16 participants; 33.33%) and least was 20–30 years (6 participants; 12.50 %). The most populous BMI category was the normal-weight group (19 participants; 39.58%). The BMI group below normal weight included 1 person (2.08%). The other participants were above normal weight. BMI classification was based on WHO (2021). Most participants had at least Senior High School level education (26 participant; 54.17%). Some worked as farmers (14 participants; 29.17%) but the largest number of participants were housewives (15 participants; 31.25%).

**Table 1.** Distribution of respondents based on the betel chewing habit of the Batak-Karo tribe in Karo District. T2DM=type 2 diabetes mellitus.

	n	Percentage (%)
**Study group** - Non T2DM without betel habit - Non T2DM with betel habit - T2DM without betel habit - T2DM with betel habit	12 12 12 12	25 25 25 25
**Gender** - Men - Women	11 37	22.92 77.08
**Age** - 20–30 - 31–40 - 41–50 - 51–60 - 61–70	6 8 9 16 9	12.50 16.67 18.75 33.33 18.75
**Body mass index** - Under weight - Normal weight - Overweight - Obese Class-I - Obese Class-II - Obese Class-III	1 19 17 6 4 1	2.08 39.58 35.42 12.5 8.33 2.08
**Duration of T2DM** - >10 years - <10 years - Never	3 21 24	6.25 43.75 50
**Betel Frequency** - Often >10 x in daily - Sometimes/rarely < 10 x in daily - Never	14 10 24	29.17 20.83 50
**Duration of Betel habit** - >10 years - <10 years - Never	12 12 24	25 25 50
**Aims of betel** - Calms the mind (mood), make the teeth be healthy and strong - Only as habit/tradition - Never	16 8 24	33.33 16.67 50
**Effects of betel** (felt comfort, change coloring teeth, fresh condition feeling, irritation in mouth, red lips, etc) - Yes - No	24 24	50 50
**Education** - No School - Elementary school - Junior high school - Senior high school - Academic - Undergraduate - Magister	2 5 5 26 4 5 1	4.17 10.42 10.42 54.17 8.33 10.42 2.08
**Occupation** - Retired/pension - Housewife/no work - Entrepreneur/sales - Civil employee - Private employee - Farmer	1 15 9 5 4 14	2.08 31.25 18.75 10.42 8.33 29.17

Table 2 shows the BGL of participants based on with/without betel habits in the non- T2DM and T2DM groups.

**Table 2.** Blood glucose levels (BGL) after fasting and 2 hours postprandial (2hPP).

Groups	BGLfasting (mg/dl)	BGL 2hPP (mg/dl)	Gap of BGL (mg/dl)	p- value
I. Non-T2DM participants without betel habit	84.33 ± 12.32	111.25 ± 22.62	26.92	0.004 *
II. Non-T2DM participants with betel habit	81.00 ± 4.84	108.33 ± 18.99	27.33	0.0001 *
III.T2DM participants without betel habit	196.25 ± 104.81	314.92 ± 128.97	118.67	0.002 **
IV.T2DM participants with betel habit	150.00 ± 42.45	229.25 ± 58.26	79.25	0.0001 *

*paired *t-test*; **Wilcoxon test; p-value <0.05 level of significance; T2DM = type 2 diabetes mellitus

This study showed that the fasting BGL and BGL changes in each group (I, II, III, and IV) there were significantly different (p<0.05). In group-I the BGL fasting (84.33 ± 12.32) mg/dl compared BGL 2hPP (111.25 ± 22.62) mg/dl, p = 0.004, whereas gap of BGL (26.92) mg/dl; group-II the BGL fasting (81.00 ± 4.84) mg/dl compared BGL 2hPP (108.33 ± 18.99) mg/dl, p = 0.0001, whereas gap of BGL (27.33) mg/dl; group-III BGL fasting (196.25 ± 104.81) mg/dl compared BGL 2hPP (314.92 ± 128.97) mg/dl, p = 0.002, whereas gap of BGL (118.67) mg/dl; and group-IV BGL fasting (150.00 ± 42.45) mg/dl compared BGL 2hPP (229.25 ± 58.26) mg/dl, p = 0.0001 with gap of BGL (79.25) mg/dl (see Table 2).

This study showed that for comparison between BGL fasting and 2 hPP in each group, there was a significant difference between group-I (84.33 ± 12.32 vs 111.25 ± 22.62; p = 0.004); group-II (81.00 ± 4.84 vs 108.33 ± 18.99; p = 0.0001); group-III (196.25 ± 104.81 vs 314.92 ± 128.97; p = 0.002); and group-IV (150.00 ± 42.45 vs 229.25 ± 58.26; p = 0.0001) (see Table 2).

Table 2 showed the differences in the gap of BGL in each group and the magnitude of the increase in BGL fasting compared to BGL 2 hours postprandial. The difference in increase in BGL groups-I and -II were almost the same, namely 26.92 mg/dl and 27.33 mg/dl, not exceeding normal glucose levels both fasting and 2 hours postprandial conditions with/without habit betel. But the highest BGL difference was found in group III-T2DM without betel habits, which was 118.67 mg/dl. Meanwhile, in the T2DM with betel habits (group-IV), the gap between BGL fasting and BGL2hPP was only 79.25 mg/dl. This indicates that the betel habit can restrain the increase in BGL as seen in groups-IV compared to group-III.

Based on the descriptive analysis obtained, in the fasting BGL condition, the average (mean) blood glucose score of group-I was 84.33. The second group-II who had the habit of betel had a blood glucose score of 81.00 for fasting BGL. The blood glucose level in group-III was 196.25, while in group-IV who had betel habits had a blood sugar score of 150. The lowest blood sugar levels (minimum) were in group-I with a BGL score of 61.00. However, the highest blood sugar levels (maximum) were in group-III with a BGL score of 420. The smallest range was in group-II with a score of 14 while the largest range obtained was in group-III with a score of 347. The standard deviation is in group-I with a magnitude of 12.32 is the nethermost while the largest is in group-III with a magnitude of 104.81 (see Table 3).

**Table 3.** Descriptive data Blood Glucose Levels fasting and 2 hours postprandial between group-I, group-II, group-III, and group-IV.

Group	BGL fasting	2hPP BGL
**I. Non-T2DM participants without betel habit** - Mean - SD - Median - Range - Minimum - Maximum	84.33 12.32 86.00 44.00 61.00 105.00	111.25 22.62 107.5 78.00 84.00 162.00
**II. Non-T2DM participants with betel habit** - Mean - SD - Median - Range - Minimum - Maximum	81.00 4.84 81.50 14.00 74.00 88.00	108.33 18.99 112.00 50.00 81.00 131.00
**III. T2DM participants without betel habit** - Mean - SD - Median - Range - Minimum - Maximum	196.25 104.81 169.50 347.00 73.00 420.00	314.92 128.97 273.00 410.00 108.00 518.00
**IV. T2DM participants with betel habit** - Mean - SD - Median - Range - Minimum - Maximum	150.00 42.45 147.00 115.00 94.00 209.00	229.25 58.26 221.50 184.00 152.00 336.00

While in the 2hPP BGL condition, the average blood glucose level of group-I was 111.25, while the average of group-II was 108.33. Both groups showed normal blood glucose levels. In the group with T2DM without betel habit (-III) the average score of blood glucose levels is 314.92, while in the group with betel habit -(IV) it is 229.25. This shows that these two groups have an average blood glucose level above normal. The lowest blood glucose level in this condition was in group-II with a score of 81.00 and the highest in group-III with a score of 518.00. The range of blood glucose levels was the lowest in group-II with a score of 50.00 and the highest in group-III with a score of 410.00. The lowest standard deviation was 18.99 in group I and the highest in group-III with 128.97 (see Table 3).

**Table 4.** Comparison of Blood Glucose Levels (BGL) fasting between group-I vs group-II and group-III vs group-IV.

	Group – I (Non-T2DM participants without betel habit)	Group – II (Non-T2DM participants with betel habit	Gap	*p*-value
**Mean**	84.33 ± 12.32	81.00 ± 4.84	3.33	0.397 *
	Group -III.T2DM participants without betel habit)	Group – IV (T2DM participants with betel habit)	Gap	*p*-value
**Mean**	196.25 ± 104.81	150.00 ± 42.45	46.25	0.378 #

*Independent sample t-test, ^#^Mann-Whitney, p-value <0.05 level of significance

**Table 5.** Comparison of Blood Glucose Levels (BGL) 2hPP between group-I vs group-II and group-III vs group-IV.

	Group – I (Non-T2DM participants without betel habit)	Group – II (Non-T2DM participants with betel habit	Gap	*p*-value
**Mean**	111.25 ± 22.62	108.33 ± 18.99	2.92	0.736 *
	Group -III.T2DM participants without betel habit)	Group – IV (T2DM participants with betel habit)	Gap	*p*-value
**Mean**	314.92 ± 128.97	229.25 ± 58.26	85.67	0.089 *

*Independent sample t-test, ^#^Mann-Whitney, p-value <0.05 level of significance

## Discussion

The frequency of betel habit among participants in this study varied. In two groups (-II and -IV), 14 participants from a total of 24 participants in those groups had betel habits > 10 times a day (29.17%). The results of this study are the same as Kanapathy's study (2014), in which 14 samples of a total of 25 had betel habit frequency > 10 times a day.

In the present study, the highest percentage of participants suffering from Diabetes Mellitus (DM) for less than 10 years was 21 participants (43.75%), in contrast to the findings of Budiharto (2018), who found that 15 out of 25 people (60%) who had suffered from DM long-term and had a l betel habit for more than 10 years. The main purpose of betel habits found in this study (16 participants, 33.33%) was to get a sense of comfort and dental health. This is supported by Budiharto's research (2018) which reported that out of a total of 25 participants (13 participants, 52%) the habit of chewing betel had the same effect. In contrast, the results of other researchers showed that as many as 68% of participants experienced porous teeth and poor oral hygiene due to betel. This could be because the subjects studied did not maintain oral hygiene, or lacked the knowledge about how to maintain oral health by chewing betel ( Andriyani, 2005).

The habit of chewing betel is often found in rural areas in Karo Regency. Chewing betel is a hereditary culture that has become a tradition of the Karo tribe to this day. This is supported by research conducted by Perangin-Angin (2017) which states that the Karo people have a tradition that involves betel activities in a series of Karo customs. However, unlike the Karo people in the countryside, the Karo people in urban areas are rarely found to have the habit of chewing betel. This may be due to hygiene factors (when they chew betel, their teeth change to turn blackish red, and also differences in busy urban lifestyles whereas Karo people who live in urban areas do not have much time to gather while chewing betel together).

Betel habit generally uses a mixture of betel leaf, lime (calcium hydroxide), gambier, areca nut, sometimes with or without the addition of tobacco. Gambier is known to prevent various diseases because besides being efficacious as an anti-inflammatory, it is also a strong anti-oxidant. Pane *et al*. (2018) reported that gambier can reduce blood glucose levels in T2DM patients by increasing levels of superoxide dismutase resulting in a decrease in malondialdehyde formation and an increase in pancreatic function in producing insulin. In the present study, we suggested that the habit of chewing betel can control BGL because of the efficacy of gambier which is one of the components in betel. This might be due to the efficacy of gambier which is one of the components in betel. It has been seen in the results that there were differences BGL in each group with an increase of the rate from fasting BGL and BGL2hPP which can be compared as follows: the group-IV (T2DM group with betel habits) had a lower difference in increasing BGL(79.25) mg/dl compared to the group-III (T2DM group without betel) as much (118.67) mg/dl. In the group that has the habit of consuming betel, glucose levels are lower than the group without habit betel. We assume the gambier who consumed as part of their betel habit can bind oxidants produced by metabolism when BGL are high, thus affecting the function of the pancreas to produce insulin. It cause of the suppressed rate of increase in BGL in participants who are suffering from T2DM with betel habit. However, in the non-T2DM group (I and II), both groups with or without betel habits had BGL within normal limits.

Gambier (Uncaria gambier Roxb) which is rich in catechins plays a role in normalize of BGL ( Sugiyama, 2005). The more importance, the anti-oxidant *catechin* molecules in gambier are safe, which were identified as the main bioactive compounds in gambier ( Anggraini *et al.*, 2011). Catechins can improve diabetes and its complications by modifying oxidative stress (p<0.05) ( Pane *et al.*, 2018; Samarghandian *et al.*, 2017).

Comparing the fasting and 2 hPP BGL between groups, there was no statistically significant difference between group-I (non T2DM participants without betel habit) and group-II (non T2DM participants with betel habit); and also group-III (T2DM participants without betel habit) compared group-IV (T2DM participants with betel habit), p>0.05 (see Table 4 and Table 5). 

In the present study, the group with betel habits (II and IV) when compared to the group without betel habits (i and III) had average lower blood sugar levels in both BGL and 2hPP fasting condition. (See Table 4 and Table 5). This indicates that the betel habit will not be suppressing BGL below the normal threshold in BGL fasting and BGL 2hPP conditions. Mathew & Tadi in 2021 stated that the limit of normal BGL is 72–108 mg/dl.

The limitations of this study were that it is not easy to collect samples that have betel habits such as groups-II and -IV, due to the small number of samples in the population. In addition, in a pandemic situation, people do not want to be at in the Public Health Center for long, especially for T2DM participants with/without betel habit groups. However, every step in this study was carried out under strict health protocol procedures.

Based on the descriptive analysis obtained ( Table 3), in the condition of fasting BGL, it showed that these two groups, I and II, have normal blood glucose levels. Meanwhile, the T2DM group, namely groups III and -IV had blood glucose levels above normal blood glucose levels. While in condition 2hPP BGL, both groups-I and II, showed normal blood glucose levels but in the other groups-III and -IV has average blood glucose levels above normal BGL.

Based on Table 4 and Table 5, in fasting and 2hPP conditions, the p-value obtained between groups-I and -II were 0.397 and 0.736. Meanwhile, the p-value obtained between BGL of groups-III and -IV in fasting conditions is 0.378 and in 2hPP conditions is 0.089. There was found a large gap between group-III and group-IV in the fasting condition, the gap value was 46.25 mg/dl while in the 2hPP condition the gap value was 85.67 mg/dl. We assume that in group-III there is no gambier to suppress BGL levels, in contrast to the group that has the habit of chewing betel, it seems that BGL levels are restrained. It is recommended for future research to focus on subjects with type 2 diabetes with a larger population.

## Conclusion

This study concluded that betel habits can restrain the increase in BGL as seen in a comparison of T2DM participants with betel habit (group-IV) compared to T2DM participants without betel habit (group-III). There were a large gap between group-III and group-IV in both BGL fasting 46.25 mg/dl and BGL 2 hPP 85.67, but this wasn’t the case when comparing participants without T2DM.

## Data availability

### Underlying data

Figshare: The Effect of Betel Habits on Blood Glucose Levels in Karo ethnic community in Karo District. https://doi.org/10.6084/m9.figshare.17871911 ( Pane *et al.*, 2022).

This project contains the following underlying data:

- 2021-data.xlsx (raw data in Indonesian)
- Output.xlsx (statistical analysis)

### Extended data

Figshare: The Effect of Betel Habits on Blood Glucose Levels in Karo ethnic community in Karo District. https://doi.org/10.6084/m9.figshare.17871911 ( Pane *et al.*, 2022).

This project contains the following extended data:

- Informed Consent.pdf
- Certificate Clinical Trial Yunita Sari Pane.pdf
- ethical clearance.pdf
- QUESIONER PENELITIAN-20122021.docx (questionnaire in Indonesian)
- Lampiran-sign.docx (information sheet in Indonesian)

Data are available under the terms of the Creative Commons Zero "No rights reserved" data waiver (CC0 1.0 Public domain dedication).
