# The effect of chewing-betel habits on blood glucose levels in the *Karo* ethnic community, *Karo* district

**DOI:** 10.12688/f1000research.75763.3

**Published:** 2022-10-18

**Authors:** Yunita Sari Pane, Yetty Machrina, Nurfida Khairina Arrasyid, Mutiara Indah Sari

**Affiliations:** 1 Department of Pharmacology and Therapeutics, Faculty of Medicine, Universitas Sumatera Utara, Medan, North Sumatra, 20155, Indonesia; 2 Department of Physiology, Faculty of Medicine, Universitas Sumatera Utara, Medan, North Sumatra, 20155, Indonesia; 3 Department of Parasitology, Faculty of Medicine, Universitas Sumatera Utara, Medan, North Sumatra, 20155, Indonesia; 4 Department of Biochemistry, Faculty of Medicine, Universitas Sumatera Utara, Medan, North Sumatra, 20155, Indonesia

**Keywords:** betel, gambier, T2DM, blood glucose levels, Batak-Karo ethnics

## Abstract

**Background:** The chewing-betel habit is a hereditary tradition from the ancestors of the *Batak*- *Karo* tribe, in Indonesia. *Karo* people believe that chewing-betel habit is their unifier. The chewing-betel habit process begins with concocting a mixture of ingredients such as betel leaf, lime, gambier, areca nut, and with or without tobacco addition, then chewed slowly. Our previous study showed that gambier extracts ( *Uncaria gambier Roxb*), can reduce blood glucose levels (BGL) in type 2 diabetes mellitus (T2DM) patients. This study aimed to analyze whether the habit of chewing betel can affect BGL in the *Karo* ethnic community in the *Karo* district.

**Methods:** In total, 48 participants from the *Karo* community were divided into 4 groups (n=12 per group), namely: I. non-T2DM participants without chewing-betel habits; II. non-T2DM participants with chewing-betel habit; III. T2DM participants without chewing-betel habit and IV. T2DM participants with chewing-betel habit. The sampling technique was consecutive sampling. The data were collected by interviews and blood sampling (fasting and 2 hours postprandial (2hPP)). The collected data were analyzed by paired t-test, Independent sample t-test, Wilcoxon, and Mann-Whitney with a significance level of p-value <0.05.

**Results:** This study showed that fasting BGL condition in group-I compared to group II (84.33±12.32 vs 81.00±4.84) mg/dl and group-III compared to group-IV (196.25±104.81 vs 150.00±42.45) mg/dl had no significant difference. Also, the BGL of 2hPP condition in group-I compared to group-II (111.25 ±22.62 vs 108.33±18.99) mg/dl, and group-III compared to group-IV (314.92±128.97 vs 229.25±58.26) mg/dl, in statistically there was no difference (p>0.05). Although the data showed that group-III was higher than group-IV.

**Conclusions:** This study concludes that with or without chewing-betel habits affect blood sugar levels in type 2 diabetes subjects (Groups III and IV).

## Introduction

The chewing-betel habit is a cultural tradition of Indonesian society with one of its compositions being gambier. Gambier ( *Uncaria gambier Roxb*) is mixed with several other ingredients and then wrapped in betel leaf which is to be chewed slowly. People who chew betel regularly have their own reasons for the habit, other than taste. Chewing betel is a hereditary tradition from ancestors in the *Karo* tribe. The *Karo* people believe that chewing-betel is a unifying activity for them ( Perangin-angin, 2017).

The habit of chewing-betel is usually done 3 times a day, namely in the morning, after lunch, and at night ( Kanapathy, 2014). The habit of chewing-betel is mostly seen in women of the *Karo* tribe, but some men do it too because chewing betel is always done when meeting with relatives, colleagues, or in other social situations ( Sinuhaji, 2010). According to the data derived from interviews, chewing betel provides some benefits, such as getting the same pleasures from smoking, relaxation, and eliminating bad breath. Chewing betel has been done for generations and it is believed that it can strengthen teeth ( Flora *et al.*, 2012) as well as maintain health.

Chewing betel has been a traditional habit for a long time. But, nowadays it is getting rare to encounter it, because there has been a shift in values. However, in rural areas, traditional habits are still strongly conducted because the people there are upholding the traditions of generations ( Perangin-angin, 2017). Chewing betel is done in different ways from one country to another, and from one region to another in the same country ( Gupta & Ray, 2004). Surprisingly, the composition of betel to be chewed is relatively consistent, which consist of betel leaf, betel nut ( *Areca Catechu*), lime (calcium hydroxide), and gambier ( *Uncaria gambier Roxb*) ( Lombu, 2014).

The content of catechin polyphenols in gambier is efficacious as an anti-oxidant that may prevent various diseases, such as diabetes mellitus ( Umeno *et al.*, 2016). This case is proved in the research of Pane *et al*. (2018) which states that gambier extract is efficacious in the treatment of diabetes by increasing levels of superoxide dismutase and lowering blood glucose levels (BGL).

Based on the description above, the researchers wanted to assess whether the habit of chewing betel can affect BGL in subjects with T2DM compared to participants without T2DM, studied in the *Batak-Karo* tribe in *Karo* District.

## Methods

### Ethical considerations

This research obtained ethical approval from the Health Research Ethics Committee of the Universitas Sumatera Utara (No. 468/KEP/USU/2021). The participants gave written informed consent after receiving an explanation from the researcher regarding the research procedure they would undergo ( Pane *et al.*, 2022).

### Participants

The sample size was estimated following the data from Kawamori *et al.* (2014) for BGL fasting and BGL 2hPP, using the following formula:

* Calculation for the sample size of BGL fasting

$$\begin{array}{c} \sigma^{2}=\frac{(\text{n}1-1)S1^{2}+(\text{n}2-1)S2^{2}}{\left(n1+n2-2\right)}\\ \sigma^{2}=\frac{(88-1)107^{2}+(33-1)77^{2}}{\left(88+33-2\right)}\\ \sigma^{2}=\frac{996,063+189,728}{119}\\ \sigma =\sqrt{9,964.630}\\ \sigma =99.82 \end{array}$$

$$\text{n}=\frac{2\sigma^{2}{\left(Z_{\left(1-\frac{\alpha }{2}\right)}+Z_{\left(1-\beta \right)}\right)}^{2}}{{\left(\text{μ}1-\text{μ}2\right)}^{2}}$$

$$\text{n}=2{\left(99.82\right)}^{2}\;\frac{{\left(1.64+0.842\right)}^{2}}{(\text{−}134-{\left(-14\right)}^{2}}$$

$$\text{n}=2(9,964.03)\;\frac{{\left(2,482\right)}^{2}}{{\left(\text{−}120\right)}^{2}}$$

$$\text{n}=2(9,964.03)\;\frac{\left(6.160\right)}{\left(14,400\right)}$$

n=2(9,964.03)(0.000428)

n=8.53

n≈9participants.

* Calculation for sample the size of BGL 2hPP

$$\begin{array}{c} \sigma^{2}=\frac{(\text{n}1-1)S1^{2}+(\text{n}2-1)S2^{2}}{\left(n1+n2-2\right)}\\ \sigma^{2}=\frac{(88-1)43.6^{2}+(33-1)39.8^{2}}{\left(88+33-2\right)}\\ \sigma^{2}=\frac{(87)\;1,900.96+(32)\;1,584.04}{\left(119\right)}\\ \sigma^{2}=\frac{165,383.52+50,689.28}{119}\\ \sigma =\sqrt{1,815.74}\\ \sigma =42.61 \end{array}$$

$$\text{n}=2\sigma^{2}\;\frac{{\left(z\alpha +z\beta \right)}^{2}}{{\left(\text{μ}1-\text{μ}2\right)}^{2}}$$

$$\text{n}=2{\left(42.61\right)}^{2}\;\frac{{\left(1.64+0.842\right)}^{2}}{(\text{−}50.2-{\left(\text{−}4.2\right)}^{2}}$$

$$\text{n}=2\left(1,815.61\right)\;\frac{{\left(1.64+0.842\right)}^{2}}{(\text{−}50.2-{\left(\text{−}4.2\right)}^{2}}$$

$$\text{n}=2(1,815.61)\;\frac{6.160}{2,116}$$

n=2(1,815.61)(0.0029)

n=10.53participants

n≈11participants.

The calculation of the number of samples in the fasting BGL group is a minimum of 9 participants, while in the 2hPP BGL group is a minimum of 11 participants.

The number of samples used in this study was taken from the largest number which calculated based on sample size formula from the two groups (BGL fasting or BGL 2hPP). The largest number of samples was taken from the BGL 2hPP group i.e: 11 participants. However, to anticipate the possibility of dropped out, the number of participants is added by 10% from a total of 11 participants ≈ 12 people.

Therefore, from the 4 groups studied each consisted of 12 participants.

σ ^2^=population variance; σ=population standard deviation (SD); n=total number of samples each group (n _1_=88; n _2_=33); $Z_{\left(1-\frac{\alpha }{2}\right)}$ = value in the standard normal distribution equal to the level of significance α = 1.64; Z _(1-β)_= value in the standard normal distribution equal to the desired, β= 0.842; µ _1_=mean outcome exposed/treatment group (-134 mg/dl) for BGL fasting and (-50.2 mg/dl) for BGL 2 hPP); µ _2_=mean outcome unexposed/placebo group (-14 mg/dl) for fasting BGL and (-4.2 mg/dl) for 2hPP BGL; S _1_=SD changes from baseline treatment group (107 mg/dl) for fasting BGL and (43.6 mg/dl) for 2hPP BGL; S _2_=SD changes from baseline placebo group (77 mg/dl) for fasting BGL and (39.8 mg/dl) for 2hPP BGL. (see data from Table 2 in Kawamori *et al.*, 2014).

The participants were divided into four groups based on the feedback from the questionnaire regarding their chewing-betel habits: Group-I. Non-T2DM participants without chewing-betel habit; II. Non-T2DM participants with chewing-betel habit; III. T2DM participants without chewing-betel habit, and IV. T2DM participants with chewing-betel habit.

**Inclusion criteria**

The participants were 2 different generations of the native *Karo Batak* tribe, aged between 20–70 years.

**Exclusion criteria**

The participants were subjects with a chronic disease (complication), such as liver disease, kidney disease, cardiovascular disease, lung disease, and T2DM on insulin therapy.

The sampling technique that used is consecutive sampling. The prospective participants were approached to join the study by conducting a survey previously at the research location to see the betel habits of the *Karo* people. After that, the research team visited the local health service center ( *Puskesmas Dolat Rayat*) to find out about the health data and the betel habits of the local community. Afterward, the team was assisted by *Puskesmas* staff in collecting the participants. Furthermore, there was a time that determined at which the research team met directly and gave explanations of the aims and objectives of the researcher to research the local community. The research team explained the benefits of chewing-betel habits for health, especially to reduce blood glucose levels in T2DM patients because of the presence of gambier ( *Uncaria gambier Roxb*) as one of the components in betel which has an antioxidant effect that can reduce BGL.

### Data collection

This research was conducted from July 2021 to October 2021 at the *Puskesmas Dolat Rayat* in *Karo* District. The data collection of the subjects’ habits in this study was done via questionnaire and blood sampling to measure BGL data in one day. In the questionnaire following are the informations asked: name, age, education, occupation, history of illness, history of medicine, family history of illness, and chewing-betel habit.

Before the team taking their blood, the subjects were asked to fast (at home) from 10.00 PM to 08.00 AM (around 10 hours) the next day. The blood samples were taken at 08.00 AM straight after fasting (fasting BGL). Following this, the subjects consumed 100 grams of white bread, and 2 hours later the blood was taken again (BGL 2 hours postprandial (2hPP)).

### Procedure of taking blood for measuring Blood Glucose Levels

Blood was drawn from the participant’s fingertip and the BGL was checked using a glucometer (Family Dr® Blood Glucose Monitoring System, AGM-5135, All Medicus Co., Ltd.). The steps taken are starting with washing hands and using an alcohol swab. After the subject's finger was dry, the test strip was inserted into the glucometer. The lancing device is prepared and open the lid on the device. Furthermore, the lancet is inserted into the lancing device by unscrewing the top back and adjusting the depth of the lancing device. The cocking handle is shifted to the rear. Then the finger examined is placed firmly on the side of the other finger and the lancing device button is pressed. After that, the finger is squeezed from the palm to the fingertips to get a drop of blood. The prepared test strip is then attached to the blood sample and the glucometer will show the test value.

### Statistical analysis

The data collected were analyzed using SPSS version 20 by paired *t-test*, Independent sample *t-test*, Wilcoxon, and Mann-Whitney with a significance value of p <0.05.

## Results

The participants in this study were the *Karo* tribe from 2 generations of pure Karo natives. Out of 49 potential participants, 48 were eligible to take part ( Pane *et al.*, 2022). The characteristics of the participants' distribution based on the chewing-betel habits are as follows:

Table 1 shows there were 12 participants in each of the 4 groups studied. It was found that 14 participants (29.17%) with chewing-betel habit groups had a frequency of betel >10 times a day, and the duration of chewing-betel habit as more than 10 years compared to less than 10 years was equal (12 participants, 25% in each group). Most participants (16 people; 33.33 %) reported benefits of chewing-betel to maintain health, especially mind relaxation (mood) and extra benefits for healthy and strong teeth, while others had no special reason for chewing-betel, only to follow their customs (8 people; 16.67%). In the T2DM group, there were 21 participants (43.75%) who had chewed betel suffering from diabetes for <10 years. The participants included 11 men (22.92 %) and 37 women (77.08 %). The most populous age group was 51–60 years (16 participants; 33.33%) and the least was 20–30 years (6 participants; 12.50 %). The most populous BMI category was the normal-weight group (19 participants; 39.58%). The BMI group below normal weight included 1 person (2.08%). The other participants were above normal weight. BMI classification was based on WHO (2021). Most participants had at least Senior High School level education (26 participants; 54.17%). Some worked as farmers (14 participants; 29.17%) but the largest number of participants were housewives (15 participants; 31.25%).

**Table 1.** Distribution of respondents based on the chewing-betel habit of the *Batak-Karo* tribe in *Karo* District. T2DM=type 2 diabetes mellitus.

	n	Percentage (%)
**Study group** - Non-T2DM without chewing-betel habit - Non-T2DM with chewing-betel habit - T2DM without chewing-betel habit - T2DM with chewing-betel habit	12 12 12 12	25 25 25 25
**Gender** - Men - Women	11 37	22.92 77.08
**Age** - 20–30 - 31–40 - 41–50 - 51–60 - 61–70	6 8 9 16 9	12.50 16.67 18.75 33.33 18.75
**Body mass index** - Under weight - Normal weight - Overweight - Obese Class-I - Obese Class-II - Obese Class-III	1 19 17 6 4 1	2.08 39.58 35.42 12.5 8.33 2.08
**Duration of T2DM** - >10 years - <10 years - Never	3 21 24	6.25 43.75 50
**Betel Frequency** - Often >10 x in daily - Sometimes/rarely < 10 x in daily - Never	14 10 24	29.17 20.83 50
**Duration of Betel habit** - >10 years - <10 years - Never	12 12 24	25 25 50
**Aims of chewing-betel** - Calms the mind (mood), makes the teeth to be healthy and strong - Only as habit/tradition - Never	16 8 24	33.33 16.67 50
**Effects of betel** (feeling comfort, change coloring teeth, fresh condition feeling, irritation in the mouth, red lips, etc) - Yes - No	24 24	50 50
**Education** - No School - Elementary school - Junior high school - Senior high school - Academic - Undergraduate - Magister	2 5 5 26 4 5 1	4.17 10.42 10.42 54.17 8.33 10.42 2.08
**Occupation** - Retired/pension - Housewife/no work - Entrepreneur/sales - Civil employee - Private employee - Farmer	1 15 9 5 4 14	2.08 31.25 18.75 10.42 8.33 29.17

Table 2 shows the BGL of participants based on with or without chewing-betel habits in the Non-T2DM and T2DM groups.

**Table 2.** Blood glucose levels (BGL) after fasting and 2 hours postprandial (2hPP).

Groups	BGLfasting (mg/dl)	BGL 2hPP (mg/dl)	∆ of BGL (mg/dl)	p- value
I. Non-T2DM participants without chewing-betel habit	84.33 ± 12.32	111.25 ± 22.62	26.92	0.004 *
II. Non-T2DM participants with chewing-betel habit	81.00 ± 4.84	108.33 ± 18.99	27.33	0.0001 *
III.T2DM participants without chewing-betel habit	196.25 ± 104.81	314.92 ± 128.97	118.67	0.002 **
IV.T2DM participants with chewing-betel habit	150.00 ± 42.45	229.25 ± 58.26	79.25	0.0001 *

*paired *t-test*; **Wilcoxon test; p-value <0.05 level of significance; T2DM = type 2 diabetes mellitus

This study showed that the fasting BGL and BGL changes in each group (I, II, III, and IV) there were significantly different (p<0.05). In group-I the BGL fasting (84.33 ± 12.32) mg/dl compared BGL 2hPP (111.25 ± 22.62) mg/dl, p = 0.004, whereas interval delta (∆) of BGL (26.92) mg/dl; group-II the BGL fasting (81.00 ± 4.84) mg/dl compared BGL 2hPP (108.33 ± 18.99) mg/dl, p = 0.0001, whereas ∆ of BGL (27.33) mg/dl; group-III BGL fasting (196.25 ± 104.81) mg/dl compared BGL 2hPP (314.92 ± 128.97) mg/dl, p = 0.002, whereas ∆ of BGL (118.67) mg/dl; and group-IV BGL fasting (150.00 ± 42.45) mg/dl compared BGL 2hPP (229.25 ± 58.26) mg/dl, p = 0.0001 with ∆ of BGL (79.25) mg/dl (see Table 2).

This study showed that for comparison between BGL fasting and 2 hPP in each group, there was a significant difference between group-I (84.33 ± 12.32 vs 111.25 ± 22.62; p = 0.004); group-II (81.00 ± 4.84 vs 108.33 ± 18.99; p = 0.0001); group-III (196.25 ± 104.81 vs 314.92 ± 128.97; p = 0.002); and group-IV (150.00 ± 42.45 vs 229.25 ± 58.26; p = 0.0001) (see Table 2).

Table 2 shows the differences in the ∆ of BGL in each group and the magnitude of the increase in BGL fasting compared to BGL 2 hours postprandial. The difference in increase in BGL groups-I and -II were almost the same, namely 26.92 mg/dl and 27.33 mg/dl, not exceeding normal glucose levels both fasting and 2 hours postprandial conditions with or without habit of chewing-betel. But the highest BGL difference was found in group III-T2DM without chewing-betel habits, which was 118.67 mg/dl. Meanwhile, in the T2DM with chewing-betel habits (group-IV), the ∆ between BGL fasting and BGL2hPP was only 79.25 mg/dl. This indicates that the chewing-betel habit can restrain the increase in BGL as seen in groups-IV compared to group-III.

Based on the descriptive analysis obtained, in the fasting BGL condition, the average (mean) blood glucose score of group-I was 84.33. The second group-II with chewing-betel habit had a blood glucose score of 81.00 for fasting BGL. The blood glucose level in group-III was 196.25, while in group-IV with chewing-betel habits had a blood sugar score of 150. The lowest blood sugar levels (minimum) were in group-I with a BGL score of 61.00. However, the highest blood sugar levels (maximum) were in group-III with a BGL score of 420. The smallest range was in group-II with a score of 14 while the largest range obtained was in group-III with a score of 347. The standard deviation is in group-I with a magnitude of 12.32 is the nethermost while the largest is in group-III with a magnitude of 104.81 (see Table 3).

**Table 3.** Descriptive data of Blood Glucose Levels fasting and 2 hours postprandial between group-I, group-II, group-III, and group-IV.

Group	BGL fasting	2hPP BGL
**I. Non-T2DM participants without chewing-betel habit** - Mean - SD - Median - Range - Minimum - Maximum	84.33 12.32 86.00 44.00 61.00 105.00	111.25 22.62 107.5 78.00 84.00 162.00
**II. Non-T2DM participants with chewing-betel habit** - Mean - SD - Median - Range - Minimum - Maximum	81.00 4.84 81.50 14.00 74.00 88.00	108.33 18.99 112.00 50.00 81.00 131.00
**III. T2DM participants without chewing-betel habit** - Mean - SD - Median - Range - Minimum - Maximum	196.25 104.81 169.50 347.00 73.00 420.00	314.92 128.97 273.00 410.00 108.00 518.00
**IV. T2DM participants with chewing-betel habit** - Mean - SD - Median - Range - Minimum - Maximum	150.00 42.45 147.00 115.00 94.00 209.00	229.25 58.26 221.50 184.00 152.00 336.00

While in the 2hPP BGL condition, the average blood glucose level of group-I was 111.25, while the average of group-II was 108.33. Both groups showed normal blood glucose levels. In the group with T2DM without chewing-betel habit (-III) the average score of blood glucose levels is 314.92, while in the group with chewing-betel habit -(IV) it is 229.25. This shows that these two groups have an average blood glucose level above normal. The lowest blood glucose level in this condition was in group-II with a score of 81.00 and the highest in group-III with a score of 518.00. The range of blood glucose levels was the lowest in group-II with a score of 50.00 and the highest in group-III with a score of 410.00. The lowest standard deviation was 18.99 in group I and the highest in group-III with 128.97 (see Table 3).

**Table 4.** The comparison of Blood Glucose Levels (BGL) fasting between group-I vs. group-II, and group-III vs. group-IV.

	Group – I (Non-T2DM participants without chewing-betel habit)	Group – II (Non-T2DM participants with chewing-betel habit	∆ of BGL (mg/dl)	*p*-value
**Mean**	84.33 ± 12.32	81.00 ± 4.84	3.33	0.397 *
	Group -III.T2DM participants without chewing-betel habit)	Group – IV (T2DM participants with chewing-betel habit)	∆ of BGL (mg/dl)	*p*-value
**Mean**	196.25 ± 104.81	150.00 ± 42.45	46.25	0.378 #

*Independent sample t-test, ^#^Mann-Whitney, p-value <0.05 level of significance

**Table 5.** The comparison of Blood Glucose Levels (BGL) 2hPP between group-I vs. group-II, and group-III vs. group-IV.

	Group – I (Non-T2DM participants without chewing-betel habit)	Group – II (Non-T2DM participants with chewing-betel habit	∆ of BGL (mg/dl)	*p*-value
**Mean**	111.25 ± 22.62	108.33 ± 18.99	2.92	0.736 *
	Group -III.T2DM participants without chewing-betel habit)	Group – IV (T2DM participants with chewing-betel habit)	∆ of BGL (mg/dl)	*p*-value
**Mean**	314.92 ± 128.97	229.25 ± 58.26	85.67	0.089 *

*Independent sample t-test, ^#^Mann-Whitney, p-value <0.05 level of significance

## Discussion

The frequency of chewing-betel habits among participants in this study is varied. In two groups (-II and -IV), 14 participants from a total of 24 participants in those groups had chewing-betel habits > 10 times a day (29.17%). The results of this study are the same as Kanapathy's study (2014), in which 14 samples of a total of 25 had chewing-betel habit frequency > 10 times a day.

In the present study, the highest percentage of participants suffering from Diabetes Mellitus (DM) for less than 10 years was 21 participants (43.75%), in contrast to the findings of Budiharto (2018), who found that 15 out of 25 people (60%) who had suffered from DM long-term and had a chewing-betel habit for more than 10 years. The main purpose of chewing-betel habits found in this study (16 participants, 33.33%) was to get a sense of comfort and dental health. This is supported by Budiharto's research (2018) which reported that out of a total of 25 participants (13 participants, 52%) the habit of chewing betel had the same effect. In contrast, the results of other researchers showed that as many as 68% of participants experienced porous teeth and poor oral hygiene due to betel. This could be because the subjects studied did not maintain oral hygiene, or lacked the knowledge about how to maintain oral health by chewing betel ( Andriyani, 2005).

The habit of chewing-betel is often found in rural areas in *Karo* Regency. Chewing betel is a hereditary culture that has become a tradition of the *Karo* tribe to this day. This is supported by research conducted by Perangin-Angin (2017) which states that the *Karo* people have a tradition that involves betel activities in a series of *Karo* customs. However, unlike the *Karo* people in the countryside, the *Karo* people in urban areas are rarely found to have the habit of chewing betel. This may be due to hygiene factors (chewing betel causing teeth turn blackish and red) in busy urban lifestyles where *Karo* people who live in urban areas do not have much time to gather while chewing betel together.

Chewing-betel habit generally uses a mixture of betel leaf, lime (calcium hydroxide), gambier, and areca nut, sometimes with or without the addition of tobacco. Gambier is known to prevent various diseases because it is both anti-inflammatory and a strong antioxidant. Pane *et al*. (2018) reported that gambier can reduce blood glucose levels in T2DM patients by increasing levels of superoxide dismutase resulting in a decrease in malondialdehyde formation and an increase in pancreatic function in producing insulin. In the present study, we suggested that the habit of chewing betel can control BGL because of the efficacy of gambier which is one of the components in betel. It has been seen in the results that there were differences in BGL for each group with an increase in the rate from fasting BGL and BGL2hPP which can be compared as follows: the group-IV (T2DM group with chewing-betel habit) had a lower difference in increasing BGL(79.25) mg/dl compared to the group-III (T2DM group without chewing-betel habit) as much (118.67) mg/dl. In the group with chewing-betel habit, the glucose levels are lower than in the group without habit of chewing-betel. We assume the gambier is consumed as part of their chewing-betel habit can bind oxidants produced by metabolism when BGL are high, thus affecting the function of the pancreas to produce insulin. It cause the suppressed rate of increase in BGL in participants who are suffering from T2DM with chewing-betel habit. However, in the non-T2DM group (I and II), both groups with or without chewing-betel habits had BGL within normal limits.

Gambier ( *Uncaria gambier Roxb*) which is rich in catechins plays a role in normalizing BGL ( Sugiyama, 2005). The anti-oxidant *catechin* molecules in gambier are safe, and identified as the main bioactive compounds in gambier ( Anggraini *et al.*, 2011). Catechins can improve diabetes and its complications by modifying oxidative stress (p<0.05) ( Pane *et al.*, 2018; Samarghandian *et al.*, 2017).

Comparing the fasting and 2 hPP BGL between groups, there was no statistically significant difference between group-I (non-T2DM participants without chewing-betel habit) and group-II (non-T2DM participants with chewing-betel habit); and also group-III (T2DM participants without chewing-betel habit) compared to group-IV (T2DM participants with chewing-betel habit), p>0.05 (see Table 4 and Table 5). 

In the present study, the group with chewing-betel habits (II and IV) when compared to the group without chewing-betel habits (i and III) had average lower blood sugar levels in both BGL and 2hPP fasting conditions. (See Table 4 and Table 5). This indicates that the chewing-betel habit will not suppress BGL below the normal threshold in BGL fasting and BGL 2hPP conditions. Mathew & Tadi in 2021 stated that the limit of normal BGL is 72–108 mg/dl.

This study has some limitations. First, a small number of samples from people with chewing-betel habits. Second, the pandemic situation causes people to not want to be at Public Health Center for a long time, especially T2DM participants with or without chewing-betel habits. However, every step in this study was carried out under the strict health protocol procedures.

Based on the descriptive analysis obtained ( Table 3), the condition of fasting BGL shows that group I and II have normal blood glucose levels. The T2DM groups namely group-III and -IV have blood glucose levels above normal. While In condition 2hPP BGL both group-I and -II shows normal blood glucose levels. But, group III and IV have average blood glucose levels above normal BGL.

Based on Table 4 and Table 5, in fasting and 2hPP conditions, the p-value obtained between groups-I and -II were 0.397 and 0.736. Meanwhile, the p-value obtained between BGL of groups-III and -IV in fasting conditions is 0.378 and in 2hPP conditions is 0.089. There was found a large gap between group-III and group-IV in the fasting condition, the ∆ value was 46.25 mg/dl while in the 2hPP condition the ∆ value was 85.67 mg/dl. We assume that in group-III there is no gambier to suppress BGL levels, in contrast to the group that has the habit of chewing betel, it seems that BGL levels are restrained. It is recommended for future research to focus on subjects with type 2 diabetes with a larger population.

## Conclusion

In conclusion, chewing-betel habit clinically inhibits the increase in BGL of T2DM patients, although it is not statistically significant different. It is showed by the different in BGL between after fasting and 2hPP of the participants with chewing-betel habit is lower than those without chewing-betel habit.

## Data Availability

Figshare: The Effect of Betel Habits on Blood Glucose Levels in Karo ethnic community in Karo District. https://doi.org/10.6084/m9.figshare.17871911 ( Pane *et al.*, 2022). This project contains the following underlying data: 2021-data.xlsx (raw data in Indonesian) Output.xlsx (statistical analysis) Figshare: The Effect of Betel Habits on Blood Glucose Levels in Karo ethnic community in Karo District. https://doi.org/10.6084/m9.figshare.17871911 ( Pane *et al.*, 2022). This project contains the following extended data: Informed Consent.pdf Certificate Clinical Trial Yunita Sari Pane.pdf ethical clearance.pdf QUESIONER PENELITIAN-20122021.docx (questionnaire in Indonesian) Lampiran-sign.docx (information sheet in Indonesian) Data are available under the terms of the Creative Commons Zero "No rights reserved" data waiver (CC0 1.0 Public domain dedication).
