# Supplementary material for: The effect of chewing-betel habits on blood glucose levels in the Karo ethnic community, Karo district
Source: F1000Res. 2023 Dec 6;11:100. Originally published 2022 Jan 26. [Version 4] doi: 10.12688/f1000research.75763.4 (PMC12624264; doi:10.12688/f1000research.75763.4)
Supplement: Peer review history

# Reviewer report: Reviewer response for version 4

**DOI:** 10.5256/f1000research.158368.r433581

**Published:** 2025-12-24

**Authors:** Reggie Surya

**Affiliations:** 1 Bina Nusantara University, Jakarta,, Indonesia

This study investigates the effect of chewing-betel habits on fasting and postprandial blood glucose levels among the Karo ethnic community in North Sumatra, Indonesia. The topic is culturally significant and addresses a relatively unexplored area of ethnomedicine, adding value to public health research rooted in a unique cultural context. The study design is logical and provides a basis for comparison. The findings suggest a possible trend toward lower BGL increase among T2DM participants with chewing-betel habits, but this is not supported by statistical significance. Clearer justification of methodology, improved coherence in the discussion, and more cautious interpretation of results would strengthen the manuscript. Please kindly find the following comments/suggestions/questions for manuscript improvement:

 1. The title should include a geographical element to clearly indicate where the study was conducted. This will enhance clarity and contextual relevance for international readers.

 2. The background section is not sufficiently strong. The authors should clearly articulate why this study was conducted, including the public health relevance and the knowledge gap it aims to address. If possible, the introduction should also discuss known biochemical or physiological mechanisms linking betel chewing (particularly gambier-derived catechins) to blood glucose regulation.

 3. The sample size calculation (page 3) is overly lengthy and contains unnecessary detail. It would be more concise to state the formula used (with citation) and present the final calculated sample size, rather than showing every step of the computation.

 4. Methodology: (a) The manuscript should explicitly describe the recruitment criteria for participants with T2DM, including how diagnosis was confirmed and whether medication status (oral hypoglycemics, insulin use, etc.) was considered. (b) The authors must clarify how food intake was controlled during BGL measurements. Different foods cause different glycemic responses; therefore, information such as the glycemic index, calorie content, or standardization of meal composition across groups is essential.

 5. Table 1 should be reorganized per study group, rather than combining all participants. Important variables such as mean BMI and mean age per group should be added, as these are critical confounding factors in glycemic studies.

 6. Tables 2 and 3 present largely the same information. Please merge them into a single, consolidated table to avoid redundancy and improve readability.

 7. The conclusion should be rewritten for clarity. The current phrasing is awkward and suggests causal effects that are not statistically supported. It is sufficient to state that no significant differences were found between groups, despite the tendency.

 8. The manuscript requires careful attention to English language usage. Several sentences are grammatically incorrect or phrased awkwardly, and overall clarity must be improved to meet international publication standards. The authors could use a language editing service or have the manuscript reviewed by a native English speaker.

 Thank you.

Is the work clearly and accurately presented and does it cite the current literature?

Partly

If applicable, is the statistical analysis and its interpretation appropriate?

I cannot comment. A qualified statistician is required.

Are all the source data underlying the results available to ensure full reproducibility?

Partly

Is the study design appropriate and is the work technically sound?

Yes

Are the conclusions drawn adequately supported by the results?

Yes

Are sufficient details of methods and analysis provided to allow replication by others?

Partly

Reviewer Expertise:

Food Secience, Food Technology, Nutrition

I confirm that I have read this submission and believe that I have an appropriate level of expertise to confirm that it is of an acceptable scientific standard, however I have significant reservations, as outlined above.

# Reviewer report: Reviewer response for version 4

**DOI:** 10.5256/f1000research.158368.r423855

**Published:** 2025-11-17

**Authors:** Biswajit Patra

**Affiliations:** 1 Fakir Mohan University, Balasore, Odisha, India

The authors have introduced a novel application of statistical modeling in betel vine research, which enhances the scientific rigor of the study. However, the following points could help strengthen the manuscript further:

1. **Introduction:**
  - Provide more background on the biochemical mechanisms that may link betel chewing to changes in blood glucose levels.
  - Clarify the specific knowledge gap this study aims to fill.
2. **Methods:**
  - Include more detail on participant selection criteria (e.g., inclusion/exclusion factors).
  - Specify how confounding factors such as diet, physical activity, or other lifestyle habits were controlled or accounted for.
3. **Results:**
  - Present more detailed summary statistics (e.g., means, standard deviations, confidence intervals).
  - If possible, provide effect sizes to show the magnitude of differences between groups.
4. **Discussion:**
  - Expand on how the findings compare with previous studies on betel chewing and metabolic outcomes.
  - Discuss possible biological explanations for the observed effects.
  - Acknowledge limitations, such as sample size or potential recall bias.
5. **Conclusion:**
  - The conclusion is concise and clear; however, the authors could briefly suggest practical implications or public health recommendations based on their findings.

Overall, this manuscript provides a valuable contribution to understanding the potential health impacts of betel chewing in the Karo ethnic community. With minor revisions and expanded discussion in key sections, it could be a strong candidate for indexing.

Is the work clearly and accurately presented and does it cite the current literature?

Yes

If applicable, is the statistical analysis and its interpretation appropriate?

Yes

Are all the source data underlying the results available to ensure full reproducibility?

Yes

Is the study design appropriate and is the work technically sound?

Yes

Are the conclusions drawn adequately supported by the results?

Yes

Are sufficient details of methods and analysis provided to allow replication by others?

Yes

Reviewer Expertise:

Life Science (Botany)

I confirm that I have read this submission and believe that I have an appropriate level of expertise to confirm that it is of an acceptable scientific standard.

# Reviewer report: Reviewer response for version 3

**DOI:** 10.5256/f1000research.139203.r208629

**Published:** 2023-10-19

**Authors:** Hadeer Akram Abdulrazzaq Al-Ani

**Affiliations:** 1 Department of Public Health, School of Medicine, University of California Davis, Davis, CA, USA

Thanks for allowing me to review this manuscript. Several corrections should be made.

1. Abstract: Please delete and rephrase the sentence “Our previous study showed that”.
2. Methodology: The sample size - Please justify why you selected this formula. Why did you select the numbers for each parameter (for example, you chose 88 for n1 and the same for others)? Justify and support with references.
3. Limitations: Please add to the limitations the accuracy of using the Family Dr® Blood Glucose Monitoring System, AGM-5135, All Medicus Co., Ltd..
4. Statistical analysis: There is a significant limitation of using independent t-tests, etc., because these tests are biased, especially when measuring the overall effects of the groups. This is very clear in the statistical analysis and interpretations obtained in this study, where there was no significance in some results while significant in others. The best statistical test should be used regardless of the sample size, normality, and others should be repeated measured with ANOVA and post-hoc because the effect size will be clearer to the readers and give the final decision on whether chewing Betel has a significant effect. Therefore, I recommend re-analyzing the statistical tests and reformatting and re-editing the manuscript. The main reason I request this is that there is no point in your study in this figure; the readers now see that chewing Betel is significant and not significant, which one should be recommended?
5. Please update your references with ones from the last five years.
6. In the discussion, please do not discuss the confounding factors like duration of use because there are no findings in your statistical outputs about it.

Is the work clearly and accurately presented and does it cite the current literature?

Partly

If applicable, is the statistical analysis and its interpretation appropriate?

No

Are all the source data underlying the results available to ensure full reproducibility?

Yes

Is the study design appropriate and is the work technically sound?

Yes

Are the conclusions drawn adequately supported by the results?

Partly

Are sufficient details of methods and analysis provided to allow replication by others?

Partly

Reviewer Expertise:

Clinical Pharmacy, Public Health, Biostatistics, Pharmacology genetics

I confirm that I have read this submission and believe that I have an appropriate level of expertise to confirm that it is of an acceptable scientific standard, however I have significant reservations, as outlined above.

# Response

**Published:** 2023-10-31

**Authors:** Yunita Sari Pane

**Affiliations:** Universitas Sumatera Utara, Indonesia

Dear Reviewer,

 Kindly check on the progress on latest revision from the 2nd reviewer for the further process.

 Thank You.

 From,

 Yunita Sari Pane

# Response

**Published:** 2023-12-10

**Authors:** Yunita Sari Pane

**Affiliations:** Universitas Sumatera Utara, Indonesia

Dear  Hadeer Akram Abdulrazzaq Al-Ani  (the second reviewer)

 Thank you for your comment on improving our article.

 Here I respond to your comment and question.

 Suggestion No. 1:

 Abstract: Please delete and rephrase the sentences “Our previous study showed that”

 Replied No. 1:

 Delete

**Question No. 2:**

 Methodology: The sample size - Please justify why you selected this formula. Why did you select the numbers for each parameter (for example, you chose 88 for n1 and the same for others)? Justify and support with references.

**Answer No.2:**

 Based on Kawamori et al., 2014 (page76 Table 2 Changes in glycemic control)

**BGL Fasting:**

 n1=92(baseline intervention group)

 n2=36(baseline placebo group)

 S1=25.8(Standard deviation of BGL fasting changes from baseline intervention group)

 S2=15.1(Standard deviation of BGL fasting changes from the baseline placebo group)

 m1=-29.2

 m2=-3.4

 Zα=1.64

 Zβ=0.842

 σ 2 == (n1- 1) S12 + (n2 - 1) S 22

 (n1 + n2 - 2)

 Thus, σ for BGL fasting is 20.43

 Thus, n for BGL fasting is 7.7≈8 subject.

**BGL 2hour PP:**

 n1=88(baseline intervention group)

 n2=33(baseline placebo group)

 S1=43.6(Standard deviation of BGL fasting changes from baseline intervention group)

 S2=39.8(Standard deviation of BGL fasting changes from the baseline placebo group)

 m1=-50,2

 m2=-4,2

 Zα= 1.64

 Zβ= 0.842

 σ 2 == (n1 - 1) S12 + (n2 - 1)S22

 (n1 + n2 - 2)

 Thus, σ for BGL 2hour PP is 42.77

 Thus, n for BGL 2hour PP is 10.57≈ 11 subject.

 From the results of the sample size formula calculation above, the sample size (n) for BGL fasting is a minimum of 8 subjects, while n for BGL2hour PP is a minimum of 11 subjects.

 Considering the possibility of sample dropout increasing by 10% from the n obtained, it was decided to take the largest sample (11 subjects), so that the number of n used in this study for each group was 12 subjects.

**Suggestion No.3:**

 Limitations: Please add to the limitations the accuracy of using the Family Dr® Blood Glucose Monitoring System, AGM-5135, All Medicus Co., Ltd.

**Replied No. 3:**

 Thirdly, limitations for accuracy of using the Family Dr® Blood Glucose Monitoring System, AGM-5135, All Medicus Co., Ltd is due to the peripheral blood(end of the extremities) is used in the study where the reading might slightly be different compared to the blood taken from a vein. Unfortunately, the study was held during the pandemic thus the situation was not favorable and caused us to carry out a simple method in the study.

**Question No. 4:**

 1. Statistical analysis: There is a significant limitation of using independent t-tests, etc., because these tests are biased, especially when measuring the overall effects of the groups. This is very clear in the statistical analysis and interpretations obtained in this study, where there was no significance in some results while significant in others. The best statistical test should be used regardless of the sample size, and normality, and others should be repeatedly measured with ANOVA and posthoc because the effect size will be clearer to the readers and give the final decision on whether chewing Betel has a significant effect. Therefore, I recommend re-analyzing the statistical tests and reformatting and re-editing the manuscript. The main reason I request this is that there is no point in your study in this figure; the readers now see that chewing Betel is significant and not significant, which one should be recommended?

**Answer No. 4:**

 Our reason for using paired t-test, Independent sample t-test, Wilcoxon, and Mann-Whitney, is because:

 1. The sample size is too small in this study and the standard deviation (SD) is too wide, especially in groups III and IV. Normally, in this case, it is a parametric statistic but the data is not homogenous, so it is not recommended to use the ANOVA test although the data in the study is parametric.

 We would like to analyze the data before and after the study is conducted, as we want to observe the difference in the BGL in each group and compare it among the groups themselves.

**Suggestion No. 5:**

 Please update your references with ones from the last five years.

**Replied No. 5:**

 Great apologies, as we can’t change the references as those have been fundamental for my study, however I consider your suggestion by providing some latest references which are realatable to my study.

 Daryanti F, Jazuli M, Florentinus TS, Hartono. Values of Character Education in the Sirih Pinang Symbol: A Cultural Value of Coastal Society. Jurnal Pendidikan Progresif. 2020; 10(2): 292-29

 Musdja. Antioxidant Activity of Cathecins Isolate of Uncaria Gambier ROXB in Male Rats. LIFE: International Journal of Health and Life-Sciences. 2018; 4(2): 34-46.

**Question No. 6:**

 In the discussion, please do not discuss confounding factors like duration of use because there are no findings in your statistical outputs about it.

**Answer No. 6:**

 We include duration in our study because we consider it as baseline data in our study, thus it is important to be measured, but not required to be included in the statistic analysis.

# Reviewer report: Reviewer response for version 3

**DOI:** 10.5256/f1000research.139203.r153576

**Published:** 2022-10-26

**Authors:** Mustofa Mustofa

**Affiliations:** 1 Department of Pharmacology and Therapy, Faculty of Medicine, Public Health and Nursing (FK-KMK), Universitas Gadjah Mada, Yogyakarta, Indonesia

Revisions have been conducted by the authors. Some suggestions have been followed.

Is the work clearly and accurately presented and does it cite the current literature?

Partly

If applicable, is the statistical analysis and its interpretation appropriate?

Partly

Are all the source data underlying the results available to ensure full reproducibility?

Partly

Is the study design appropriate and is the work technically sound?

Partly

Are the conclusions drawn adequately supported by the results?

Partly

Are sufficient details of methods and analysis provided to allow replication by others?

Yes

Reviewer Expertise:

Pharmacology, drug discovery and development.

I confirm that I have read this submission and believe that I have an appropriate level of expertise to confirm that it is of an acceptable scientific standard.

# Reviewer report: Reviewer response for version 2

**DOI:** 10.5256/f1000research.137109.r147870

**Published:** 2022-09-05

**Authors:** Mustofa Mustofa

**Affiliations:** 1 Department of Pharmacology and Therapy, Faculty of Medicine, Public Health and Nursing (FK-KMK), Universitas Gadjah Mada, Yogyakarta, Indonesia

I found the authors have made revisions, however, the revisions are not enough appropriate. Therefore, I suggest the authors should make revisions again. English grammar and terms should be revised again. For example, "betel habits" maybe can be revised to be "chewing-betel habits". I also found some scientific terms are not appropriate. For example "gap", it is not a scientific term. Maybe "∆" (delta) is more appropriate. Conclusion could be presented based on statistical results and clinical results.

Is the work clearly and accurately presented and does it cite the current literature?

Partly

If applicable, is the statistical analysis and its interpretation appropriate?

Partly

Are all the source data underlying the results available to ensure full reproducibility?

Partly

Is the study design appropriate and is the work technically sound?

Partly

Are the conclusions drawn adequately supported by the results?

Partly

Are sufficient details of methods and analysis provided to allow replication by others?

Yes

Reviewer Expertise:

Pharmacology, drug discovery and development.

I confirm that I have read this submission and believe that I have an appropriate level of expertise to confirm that it is of an acceptable scientific standard, however I have significant reservations, as outlined above.

# Response

**Published:** 2022-09-13

**Authors:** Yunita Sari Pane

**Affiliations:** Universitas Sumatera Utara, Indonesia

Dear Prof. Mustofa,

 We really appreciate your willingness to review my manuscript.

 We will correct it as per your suggestion.

 Thanks for your work in improving this article.

 Best regards,

 Yunita Sari Pane

# Reviewer report: Reviewer response for version 1

**DOI:** 10.5256/f1000research.79681.r121181

**Published:** 2022-04-12

**Authors:** Mustofa Mustofa

**Affiliations:** 1 Department of Pharmacology and Therapy, Faculty of Medicine, Public Health and Nursing (FK-KMK), Universitas Gadjah Mada, Yogyakarta, Indonesia

1. The manuscript needs extensive revision for language and grammar. Some editing is required.

 2. The inclusion criteria used are not presented specifically.

 3.  The methods sections normally presented in narration not in instruction.

 4. The statistical analysis especially between group III and IV needs to be checked again:

- The main objective of the study was to evaluate the effect of betel habits on BGL. Therefore, analysis and discussion should be focused by comparing the results of groups III and IV. The sample size is too small in this study and the standard deviation (SD) is too wide, especially groups III and IV. Normally, in this case, a nonparametric statistic is recommended. In this study, the Anova was applied. Therefore, I recommend using nonparametric statistics with data presented in median +/- range.

5. The conclusion needs revision:

- It was too early to conclude that betel habits can restrain the increase of BGL. The authors did not compare the fasting BGL between group III and group IV. The conclusion just based on the BGL 2 h PP. Moreover, the conclusion should be revised based on the new statistical analysis results.

Other:

- The quality of the manuscript should be improved. For example, it is needed to present the calculation of the sample size in this manuscript. The English also should be improved.

Is the work clearly and accurately presented and does it cite the current literature?

Partly

If applicable, is the statistical analysis and its interpretation appropriate?

Partly

Are all the source data underlying the results available to ensure full reproducibility?

Partly

Is the study design appropriate and is the work technically sound?

Partly

Are the conclusions drawn adequately supported by the results?

Partly

Are sufficient details of methods and analysis provided to allow replication by others?

Yes

Reviewer Expertise:

Pharmacology and Ethnopharmacology.

I confirm that I have read this submission and believe that I have an appropriate level of expertise to confirm that it is of an acceptable scientific standard, however I have significant reservations, as outlined above.

# Response

**Published:** 2022-08-02

**Authors:** Yunita Sari Pane

**Affiliations:** Universitas Sumatera Utara, Indonesia

Thank you for reviewing and suggesting some constructive amendments to the article.

 Answering the question number:

1. We’ve revised the article for language, grammar, and editing.
2. Inclusion and exclusion criteria have been presented (see page 4).
3. The method section has been presented in narration (see page 4).
4. Statistical analysis of the articles has been adjusted and the discussion has focused more on groups 3 and 4 as suggested.
5. The conclusion of the article has also been fixed

- The conclusion of the article has been revised based on improved statistical analysis
- The conclusion of the sample size calculations has been presented since the inception of the publication

Thank you for your suggestion, we had been trying to improve our ability in scientific language.
